# Feasibility and acceptability of WeCare Mentoring, an online peer mentoring program for aged care support workers

**DOI:** 10.1093/geroni/igaf094

**Published:** 2025-09-10

**Authors:** Karol J Czuba, Alain C Vandal, Nicola M Kayes

**Affiliations:** Centre for Person Centred Research, Auckland University of Technology, Northcote, Auckland, New Zealand; Department of Biostatistics and Epidemiology, Faculty of Health and Environmental Sciences, Auckland University of Technology, Auckland, New Zealand; Centre for Person Centred Research, Auckland University of Technology, Northcote, Auckland, New Zealand

**Keywords:** Job satisfaction, Workforce development, Employee turnover, Work stress, Mixed-methods research

## Abstract

**Background and Objectives:**

In recognition of the aging population and aged care workforce shortages, calls have been made for responsive and effective strategies for this workforce group. This study aimed to investigate the feasibility, acceptability, and preliminary efficacy of an online mentoring program for aged care support workers serving older adults in New Zealand residential care facilities.

**Research Design and Methods:**

This mixed-methods study consisted of (1) a nonrandomized single-arm intervention study, with outcome measurement at baseline, 3- and 6-month (Satisfaction with Life Scale, Generic Job Satisfaction, Perceived Stress Scale, and General Self-Efficacy Scale) and (2) a post-intervention qualitative descriptive study exploring perceived acceptability and feasibility aspects of the proposed intervention. Participants met once a month, for 30–60 min. They followed a program manual to work on self-identified goals.

**Results:**

Thirty-eight support workers enrolled, and 22 of them took part and completed the 6-month program. The recruitment target was reached within the proposed 3-month timeframe. Data collection procedures were considered practical and convenient. Participants (13 mentees and 9 mentors) reported that the program was appealing and relevant, its duration and intensity appropriate, and the online delivery acceptable. Participants proposed refinements to improve their experience further. Exploratory outcomes analysis found all measures trended in the expected direction.

**Discussion and Implications:**

The WeCare Mentoring Program was found to be a feasible and acceptable intervention. Participants reported several improvements in their well-being and their caregiving-related skills. The next step is to test the intervention’s effectiveness in a definitive controlled trial or quasi-experimental study. If future efficacy trials prove successful, this program can offer a much-needed support to the aged care workforce, and lead to better outcomes for them and the people they care for.

Innovation and Translational Significance This study demonstrated the feasibility, acceptability, and preliminary efficacy of an online mentoring intervention for aged care support workers. The findings show that it has the potential to enhance support workers’ job satisfaction, well-being, and caregiving-related skills, which are critical in the quality of residential care for aging populations. Improved support for aged care workers ultimately contributes to better care experiences, contributing to sustainable, high-quality services for older people. The next step is to test the intervention’s effectiveness in a definitive controlled trial or quasi-experimental study.

## Background

Aged care has been one of the fastest growing sectors of the care economy (Ratcheva et al., 2020). The number of people requiring aged care services is increasing, and some predictions estimate that the workforce size will need to increase fourfold to meet the projected demand (Christensen et al., 2009; Productivity Commission, 2011; Rudnicka et al., 2020). The provision and quality of aged care are largely dependent on a supply of support workers (also known as nursing assistants, healthcare aides, paid caregivers) (Ludlow et al., 2021; Willis et al., 2016). This critically important workforce has been dealing with increasingly high workloads, understaffing, high emotional demands, lack of career progression opportunities, and reports feeling inadequately supported and undervalued (Eagar, 2019; Gao et al., 2017; Hewko et al., 2015; Kovaleva et al., 2018). In recognition of the aforementioned challenges, calls have been made to develop responsive and effective strategies to grow and strengthen this workforce group (World Health Organization, 2017).

The current study was the third phase of a larger project aiming to develop an online mentoring intervention to improve outcomes for aged care support workers. The larger project was guided by the Medical Research Council framework for developing complex interventions (Craig et al., 2008). In the first phase, we completed a systematic literature review and meta-analysis of peer-led interventions to improve outcomes for support workers (Czuba et al., 2023). We found that interventions focused on enhancing the workers’ understanding of issues related to the provision of aged care were effective; however, the effect sizes were small, and the certainty of evidence was low. In the second phase of the larger project, we developed an intervention protocol and tested its usability and acceptability with a group of aged care support workers. The proposed intervention, named the WeCare Mentoring Program, was a 6-month online mentoring program. The core components were the program onboarding, mentor/mentee matching, goal setting, monthly online mentoring sessions, and self-reflection through completion of meeting reports. The specific focus of the program was determined by each mentee’s needs and preferences and could involve areas in their personal as well as their professional lives. Taking part was expected to benefit both mentees and mentors. The Phase 2 study focused specifically on testing the initial stages of the proposed process: training, matching, introduction, and the first meeting. We found that the intervention protocol was acceptable to its users (Czuba et al., 2025). Several refinements were proposed, focusing on the intervention training, clarity of intervention manuals, and program and role expectations.

The study reported herein was the third phase of the larger project. The study aimed to investigate the feasibility and acceptability of the full, 6-month online WeCare Mentoring Program. Specifically, in this study, we investigated the recruitment and onboarding protocols, data collection procedures, and participants’ preliminary response to the intervention. We also assessed the intervention fidelity, estimated the number of participants required for a future definitive study and evaluated the psychometric performance of the outcome measures to inform the selection of a primary outcome for the full study.

## Research design and methods

### Research design

This study consisted of:

1. a nonrandomized single-arm intervention study, with outcome measurement at baseline, 3- and 6-month and
2. a post-intervention qualitative descriptive study exploring perceived acceptability and feasibility aspects of the proposed intervention.

Ethical approval was obtained from the Health and Disability Ethics Committee (Ethics approval number: 18/NTA/120/AM03).

### Participants

Participants (mentees and mentors) were recruited from residential aged care facilities in New Zealand (NZ). Mentee participants were included if they were:

1. over 18 years old and
2. working as an aged care support worker.

For mentors, there were two additional criteria relating to their skills and expertise in caregiving:

1. at least 5 years of experience in aged care and
2. at least Healthcare Assistant Level 4 NZQA qualification, or equivalent.

Potential participants were excluded if they had no access to the internet or were unable to use digital devices.

Mentors and mentees were matched into pairs and took part in a 6-month mentoring intervention. We aimed to recruit 20 participants as mentees, and four to eight as mentors. A purposively selected subsample (*n* = 10; seeking diversity in demographic characteristics) took part in post-intervention qualitative interviews. The sample size was considered appropriate for this exploratory study (Orsmond & Cohn, 2015), allowing evaluation of the integrity of the intervention protocol and investigation of the study objectives.

#### Procedure

This study involved a 3-month recruitment period, a 6-month online intervention, and then one more month for completion of the qualitative interviews. Taking part required users to have access to the internet, an email account, and a Skype account. First author (K.C.) acted as the program coordinator.

As above, we aimed to recruit up to 20 mentees within the 3-month period to provide an indication of the rate of recruitment to guide the design of a future definitive study. We used three recruitment methods: (1) posters, (2) presentation and pamphlet (presented in person by K.C.), and (3) staff union email invitation (see Supplementary Methods Section 1 in Supplementary Material).

Participant screening was completed over the phone. Following confirmation of their eligibility, those who intended to partake as mentors were asked to provide a character reference confirming their suitability. Next, following consent, participants completed a demographic form, a mentor/mentee preference form, and the Ten-Item Personality Inventory (Gosling et al., 2003). Mentors were also asked to indicate the maximum number of mentees they were willing to be matched with: between one and five.

Immediately prior to the intervention, all participants underwent onboarding tailored to their role. They were given 2 weeks to familiarize themselves with the program manual (available upon request from the corresponding author), and were given extra time if needed. Next, they attended a 1-hr online onboarding session (led by K.C.), during which they discussed the manual’s content in more depth, and any outstanding questions were answered.

After completing the onboarding, mentors and mentees were matched based on their demographic characteristics, personality similarity, and preferences. Additionally, participants were only eligible for matching if they did not work at the same facility. Three mentor profiles were presented to each mentee, who were then able to make the final selection.

Following the mentor–mentee matching, mentors were tasked with arranging the first meeting. Participants were instructed to meet at least once a month, for approximately 30–60 min, over the following 6 months. They were asked to follow the instructions given to them in the program manual, with their first meeting focusing on introductions, and then moving on to setting goals and plans for achieving them. This was followed by a progression phase, where the focus was on sustaining commitment and increasing the mentee’s responsibility for managing the relationship. The last phase included the final evaluation and winding down of the mentoring relationship. After each meeting, all participants were asked to complete an online meeting report, which they shared with the researcher. At the end of the program, all participants received a certificate of completion. Finally, a subsample of participants was then qualitatively interviewed.

#### Data collection

A range of qualitative and quantitative data were collected, including administrative data related to the conduct of the study (such as the recruitment activities, duration of mentoring sessions, completion of session reports, etc.). Demographic data, such as participants’ age, gender, and ethnicity, were collected directly from the study participants using a demographics form. We also obtained deidentified demographic data (including age, gender, ethnicity, length of service, employment type, immigration status, and NZ qualification level) from all facilities where the recruitment posters and presentations were used.

#### Outcome measures

Building on (Ensher & Murphy, 2007) and our prior published work (Anonymized for Review), the following measures were used: Satisfaction with Life Scale (SWLS) (Diener et al., 1985), Generic Job Satisfaction Scale (JSS) (Macdonald & Maclntyre, 1997), Perceived Stress Scale (PSS) (Cohen et al., 1994), and General Self-Efficacy Scale (GSES) (Schwarzer & Jerusalem, 1995). For each scale, the total score is calculated by summing item scores (see Supplementary Methods Section 2 for a detailed description). Outcome measurement was completed via an online survey immediately after completing the onboarding session (before mentor–mentee matching) and 3 and 6 months later.

All four scales have been found to be valid and reliable for use in research with the general population and also a range of occupational groups, including support workers (Cohen et al., 1994; Diener et al., 1985; Macdonald & Maclntyre, 1997; Scholz et al., 2002).

### Participant interviews

After completion of the 6-month mentoring program, semi-structured interviews were conducted by K.C. (see Supplementary Methods Sections 3 and 4 for the interview guides), exploring participants’ perspectives of perceived benefit, barriers, and facilitators to engaging in a mentoring relationship, mentor–mentee matching fit, onboarding, data collection procedures, and other aspects pertaining to the feasibility of the proposed intervention. The interviews were conducted in person or online (depending on participants’ geographical location and preference) immediately following the completion of the program. They were audio-recorded and transcribed.

### Data analysis

Demographic data were analyzed descriptively (frequency, central tendency, variation, and position).

Qualitative interviews were coded using Directed Content Analysis (Hsieh & Shannon, 2005). The coding framework focused on the following prespecified categories: recruitment protocols (e.g., participants’ perspectives on the recruitment methods), data collection procedures (e.g., perspectives on the format and wording of outcome measures), the WeCare Mentoring program (e.g., perspectives on relevance of the program), and on preliminary responses to taking part (e.g., perspectives on how taking part affected the participants). Coding of all transcripts in QSR NVivo was conducted by K.C., who read the transcripts multiple times to become immersed in the data. The emerging findings were reviewed at monthly progress meetings. All authors discussed the findings and the interpretations of participants’ reports. Pseudonyms are used when reporting qualitative data.

Several quantitative indicators related to the study processes were calculated: response rate, consenting rate, follow-up rate, session completion rate, and others (Supplementary Methods Section 5).

Internal consistencies of the outcome measures at each assessment point were evaluated using Cronbach’s alpha coefficients. Pearson’s R correlations between the outcome measures at each assessment point were calculated to assess the outcome measures’ convergent and discriminant validity; estimated correlations between baseline and final outcome values are also reported. Drawing on findings from other studies (Czuba et al., 2025; Liu & Aungsuroch, 2019; Schwarzer & Jerusalem, 1995), we expected the satisfaction and self-efficacy measures to correlate with one another, and the stress measure to show very low or negative correlation with other measures.

A linear mixed model fitted all outcomes as a four-­dimensional repeated measure with a similar unstructured covariance between outcomes at each assessment and participant-specific random effects against an interaction of the outcome type and assessment time point. The main fitted contrast was the difference in each score between 6 months and baseline. The difference was tested using outcome-specific observed significance of the difference and represented as an effect size, defined as the fitted difference divided by the standard deviation of the outcome at baseline and its 95% confidence interval on a forest plot. This work was carried out using SAS/STAT software version 9.4.

Finally, participant feedback was synthesized alongside the research team’s interpretation and judgment to propose protocol changes that were considered feasible or desirable for the future definitive trial.

## Results

### Recruitment

Recruitment commenced on April 1, 2019 and lasted 3 months. We delivered 25 recruitment presentations and displayed invitation posters across 16 facilities. The invitation email from the staff union was sent out to *n* = 1,620 of its members (NB: some of the respondents were nurses or activities coordinators, not support workers), 16 days before the end of the recruitment period. No new participation requests were accepted after June 30, 2019. Overall, out of an estimated pool of 2,400 participation invitation recipients, 84 people requested further information, resulting in an estimated overall response rate of 4% (in-person presentation [7%], via poster [1%], and via union email [4%]). Table 1 presents the estimated response rates by recruitment type.

**Table 1.** Estimated response rates in Phase 3 recruitment.

Type	Number of people who responded	Estimated total number of recipients	Estimated response rate
**Via poster**	5	560	1%
**Via presentations**	15	210	7%
**Via email**	64	1,620	4%
**Combined**	84	2,400	4%

The time required to complete the necessary recruitment activities (e.g., liaising with an organization, travel to facilities, hanging up posters, presenting) per respondent was over 5 hr for posters, 2.5 hr for presentations, and 2 min for email invitations.

Out of the 84 people who were interested in taking part, the majority wanted to partake as mentees (*n *= 48). Twenty-four people stopped engaging with the recruiting researcher after their initial response. Another 22 were excluded at screening with the reasons being, for example, not working in aged care, not a support worker, or responding past the recruitment period deadline.

In total, 38 people consented to take part in the study (54.3% overall consent rate; 60% via poster, 57.1% via presentation, and 52.9% via staff union email) and are referred to as the enrolled participants. Twenty-two of them completed the mentoring program and are referred to as the study participants. The majority of participants were female and NZ citizens/residents. Table 2 presents a comparison of demographic characteristics between participants and the aged care support workforce employed at the recruitment sites.

**Table 2.** Demographic characteristics of enrolled participants and the overall workforce employed at the recruitment sites.

Demographic characteristic	Participating enrollees (*n *= 22)	Nonparticipating enrollees (*n *= 16)	Overall workforce^a^
	Mean (*SD*; min, max) or *N* (%)	Mean (*SD*; min, max) or *N* (%)	Mean (*SD*; min, max) or *N* (%)
**Age, in years**	47.3 (29; 64; 12.7)	49.6 (24; 71; 11.80)	43.6 (16; 76; 13.5)
**Year(s) of experience in aged care**	10.0 (1; 30; 7.1)	9.62 (1; 30; 7.24)	5.9 (0; 37; 7.0)^b^
**Working hours per week**	34.8 (20; 40; 5.8)	36.89 (15; 50; 6.74)	Not available
**Employment type**			
** Full time (at least 37.5 hr/week)**	11 (50.0)	10 (62.5)	199 (36.0)
** Part time/Casual (less than 37.5 hr/week)**	11 (50.0)	6 (37.5)	254 (64.0)
**Gender**			
** Female**	20 (90.9)	16 (100.0)	494 (89.7)
** Male**	2 (9.1)	0 (0.0)	57 (10.3)
**NZ immigration status**			
** Citizen/Resident**	16 (72.7)	14 (87.5)	243 (77.1)
** Work Visa**	6 (27.3)	2 (12.5)	72 (22.9)
**Participant type**			Not applicable
** Mentor**	9 (40.9)	8 (50.0)	
** Mentee**	13 (59.1)	8 (50.0)	
**Ethnicity^c^**			
** NZ European**	5 (22.7)	4 (25.0)	61 (19.4)
** Māori**	3 (13.6)	2 (12.5)	9 (2.9)
** Pacific peoples**	4 (18.2)	5 (31.2)	112 (35.6)
** Asian**	8 (36.4)	2 (12.5)	112 (35.6)
** MELAA**	2 (9.1)	2 (12.5)	11 (3.5)
** Other**	0 (0.0)	1 (6.2)	7 (2.2)

*Note*. MELAA = Middle Eastern/Latin American/African; NZ = New Zealand.

a Total numbers for each variable vary between *n *= 315 and *n *= 551.

b With the current employer.

c As per NZ stats Level 1 ethnicity reporting.

We reached the goal of obtaining consent from 20 mentees within the 3-month recruitment window (total mentees recruited *n* = 21); however, only 13 eventually took part in the program. Four withdrew due to other commitments, and the other four stopped responding to our attempts to contact them. Mean ± SD time between participants’ first response and their consent decision was 8.5 ± 4.9 days.

A subsample of *n* = 13 participants was invited to discuss several aspects of their experience in the study. Three of those approached declined to take part, explaining they were too busy. The final group included four mentors and six mentees, with a broad range of work experience, age, ethnicity, and immigration status (Table 3).

**Table 3.** Demographic characteristics of study participants who took part in qualitative interviews.

Pseudonym	Role	Years of experience	Age	Ethnicity	Immigration status
**Jenny**	Mentee	1	33	Asian	Work Visa
**Nicola**	Mentor	30	60	MELAA	Citizen/Resident
**Melinda**	Mentee	11	59	Asian	Work Visa
**Judy**	Mentor	16	62	Māori/NZ European	Citizen/Resident
**Eliza**	Mentor	9	37	Asian	Citizen/Resident
**Stacey**	Mentee	1	34	Pacific Peoples	Citizen/Resident
**Mike**	Mentee	4	33	Asian	Work Visa
**Eva**	Mentee	2	31	Asian	Work Visa
**Silvia**	Mentee	10	59	Pacific Peoples	Citizen/Resident
**Paula**	Mentor	7	42	Asian	Citizen/Resident

*Note*. MELAA = Middle Eastern/Latin American/African; NZ = New Zealand.

The majority of participants found out about the study through the staff union email. Some suggested that seeing the union’s support for the program made it more appealing and “more neutral,” allowing them to talk freely about the things they found important. They wanted to share their views safely, without their employers finding out about their specific concerns or challenges.

Paula, who found out about the study from a poster, would have preferred to attend the recruitment presentation to get a more detailed description of the program. On the other hand, Melinda argued that the recruitment method did not matter to her, as “people knew what mentoring was” and she was interested in taking part. However, she mentioned that many of her workmates were not aware of this program at all, “they had no idea.” Notably, some participants might have been exposed to more than one recruitment method.

The most commonly reported reason to sign up as a mentor was the desire to share knowledge and help others. Among those who signed up as mentees, some mentioned wanting to simply become better caregivers. Melinda argued that this program could become part of all new support workers’ induction training. Stacey, who was new to caregiving, did not think the 2-week induction training she received at her workplace was sufficient. Others had well-defined work-related goals, such as completing a Level 4 Aged Care Support Certificate, and wanted someone to support them through the process. Another aspect of the program that seemed appealing to participants was that it was not only about their work but also about their lives in general. Jenny argued that she probably would not be as interested if there were an obligation to focus only on work-related goals. Mike, who was a recent migrant, reflected:

For someone like me, who was just a beginner in New Zealand, it made me able to grasp the concept of health care assistant from those people who are experienced, like my mentor. Our topics were focusing not just on work, but a very holistic approach, which was great help for a newbie like myself. (Mike, mentee)

### The WeCare Mentoring program

As above, all mentors and mentees had to complete a brief onboarding before commencing their mentoring relationships. It consisted of two parts: reading the program manual and attending one online onboarding session with the program coordinator/researcher (K.C.). None of the participants required follow-up training sessions. Mean ± SD time between consenting to participation and completing the training was 18.0 ± 5.9 days.

Participants’ feedback suggested that the onboarding prepared them well for their roles in the program. They also appeared to feel in control of the process and not rushed. As Eva reflected, they were given enough time to study the program manual. The ability to contact the researcher to help with any potential issues appeared to be valuable to participants.

It was good to talk to you via Skype, you talked me through it, all the questions I had, you talk me through it along the way. And another thing is that I could talk to you straight away if I had a problem. It was very good to be able to talk to you straight away. To know that you will be there. (Nicola, mentor)

All participants received the program manual as part of their onboarding. It served as an introduction to the mentoring concept and a guide throughout the program. Participants’ reports suggested they found it helpful, its language clear and simple, and thought the volume was appropriate. Some used a printed copy, while others used a digital version.

Following the onboarding, participants were matched into pairs. Some mentors were matched with more than one mentee, one mentor was matched with two mentees, and one other with five mentees. Mentees appeared to enjoy working with their mentors, and most were able to connect right away. Those who took part as mentors also reported feeling their mentees were well matched. For most participants, this appeared to be a two-way relationship, where both sides were able to learn from each other.

Mentees discussed wanting mentors who were experienced caregivers, able to use real-life examples, and happy to share their expertise. Like others, Mike really valued his mentors’ expertise and how much in common they had:

What I liked about him is that he is very experienced. And health care, plus also he lived here where I live, so we have common grounds. Came from the same country, and a very similar experience to our lives in New Zealand. Dealing with immigration. I don’t have any dislikes for him. It was a very good relationship. (Mike, mentee)

Some participants mentioned that they would sometimes speak in their first language with their mentor, for example, in Te Reo Māori or Tagalog. Others argued that being able to speak the same language was not as important as having similar beliefs.

Nicola, who mentored five mentees, argued that her workload “was fine.” Nonetheless, she reported that working with one of her mentees was particularly challenging, for example, when trying to an open conversation. She later found out that this particular mentee was concerned about her privacy and “did not want her name to come up somewhere unnecessarily.”

Most mentees perceived their mentors to be well prepared and accessible. However, Melinda felt her mentor did not have the ability to help her achieve her goals. She suggested that providing mentees with more background information on potential mentors would support a more fitting match selection. Additionally, she wondered if mentees could meet the potential mentors via Skype before making the final decision.

To facilitate participants’ engagement and preparedness, they were emailed meeting plans prior to their session each month. These plans helped to focus the conversation on the mentee’s goals. Participants suggested they also helped to keep the meetings relaxed and “*flowing*,” while learning new things. She described the role these plans played for her and her mentees:

It was much easier for us, instead of browsing through the manual, to just use the plan. You were sending out the meeting plan, so we thought we might as well just do the meeting plan. Instead of looking at the programme manual. Because we were talking about our day-to-day stuff, the meeting plan was just much easier, so we were just getting on with it. (Eliza, mentor)

It was primarily the participants’ responsibility to schedule their mentoring sessions. They occurred in their private time and had to be negotiated against their other competing demands, such as family commitments or studying. While some participants scheduled all their meetings in advance, others had to schedule and reschedule each meeting due to changes in their work rosters. It appeared that most participants would have appreciated being able to schedule the sessions during their work hours, for example, as part of their professional development.

Being able to do it [during work hours] would be much better. At work, we are given 2 hours every Monday. It’s for us to study, and it is sponsored. (Stacey, mentee)

All mentees had to identify one or more goals for the program. Mentees talked about how it helped them track their progress and used it as a point of reference. For some, goals changed during the program, for example, from wanting to complete an educational course to having to focus on own well-being in response to a changing life situation. However, mentors reported they were able to adapt to the changing needs of their mentees.

Participants’ reports suggested that two key characteristics making the program acceptable and suitable were the privacy it offered and its flexible one-on-one format. They felt they could be honest and talk openly with their mentor.

They will not judge me because they are not my co-worker. Just someone who works in the same industry. And we were able to tell each other what happened and give feedback, say maybe this is something you should do or something like that. (Eva, mentee)

The online delivery of the program appeared to enhance the experience of privacy. Nicola reported that one of her mentees did not want other people to know that they were taking part in the program. They thought it could have negatively affected how they were perceived by their colleagues. For Eva, the online format allowed her to match with someone from a different workplace, which helped her be open with the mentor.

Participants also reported potential downsides to the online format. For example, Sylvia experienced internet connectivity issues, which meant she missed a mentoring session. Judy was initially quite skeptical about meeting someone via video and was worried she would miss out on the nonverbal communication. However, she found that this did not become an issue.

Some participants expressed interest in meeting with their mentoring match in person after completing the program. One of them argued that meeting in person was overall a more appropriate way of delivering this kind of a program, and she wondered whether it should be delivered via the internet at all.

Finally, during her interview, Sylvia expressed an interest in becoming a mentor. The idea of progressing from a mentee to a mentor was then presented to some of the other participants. They agreed this could work well as a progression step for mentees in the program.

### Intervention fidelity

Following the onboarding, 10 mentors and 15 mentees were matched into mentoring pairs. At this point, one mentor and two mentees withdrew, all stating they were “too busy to take part.” Another participant, who completed the training and whose mentees withdrew from the study prior to their first session, agreed to take part as a “standby” mentor and completed all outcome measures. No protocol violations or adverse events were reported.

In total, 78 mentoring sessions were scheduled and 75 were conducted (96%). Mean ± SD number of completed mentoring sessions was 5.7 ± 2.9. Eleven mentoring pairs completed all their planned sessions. One pair missed two meetings, and another pair missed one.

Participants argued that the appropriate frequency of meetings depended on each mentee and their goals. For some, meeting more frequently did not seem feasible. Others believed that a lot can happen in a month, and more frequent meetings would have been more appropriate. Nicola, who mentored five mentees, also reported that her preference would have been for more frequent meetings. Those who had more tangible goals, for example, getting a qualification, also argued for meeting more than once a month.

Participants were given a guideline to keep their meetings between 30 and 60 min. Mean ± SD session duration was 34.17 ± 10.87 min. The longest session lasted 60 min, and the shortest was 14.5 min. The proportion of sessions lasting between 30 and 60 min (as per the program guidelines) was 70.6%. Another 15 meetings (20%) lasted between 25 and 30 min.

Following each session, participants were expected to complete an online meeting report, which included providing a reflection on the progress toward achieving their goals. We found that goals were set or reviewed during nearly every session (98.6%), and the reports were completed by both mentees and mentors after every session (100%). The idea behind asking participants to complete the reports was to encourage reflection and facilitate learning new strategies or habits.

Somehow the reports helped me reflect on what happened, because it was like… every time we had a meeting, I would think of what my mentor said. And I was thinking, how would I apply this at work? Is it applicable? Or not? It was really good for reflection. (Eva, mentee)

Participants’ reports regarding the program duration were mixed. For some, 6 months seemed too long. Sylvia argued that after 3 months, everything she wanted to talk about had already been covered. For others, 6 months was appropriate, with Eva adding that she “might get too attached to her mentor, if it was going for longer.” However, a few participants reported feeling surprised when they received an email telling them they were about to finish the program, and some suggested that a 12-month duration with an ability to choose a new mentor partway through would be valuable.

My mentee has done very well, but 12 months would keep her going. Just to keep her more confident and let her stabilise, and not to fluctuate. Maybe until you get to the point where you know that she can fly on her own? I think a year is the maximum, more than a year, it could be boring. But yeah, maybe just a little bit longer. (Paula, mentor)

### Data collection and outcome measures

Participants reported the data collection process and instructions were straightforward and clear. Two participants initially experienced issues with the meeting report submissions. They both were certain that they had completed and submitted their reports, but these were not saved in the system. After resending the submission instructions to participants, those issues were resolved.

Outcome data collection follow-up rates were 100% at baseline and 3 months, with only one submitted survey not returned at 6 months (95%). Additionally, one survey (at baseline) was returned outside the 2-week baseline data collection period. None of the returned surveys had any missing data.

Participants found the measures to be relevant to the program’s objectives. Sylvia suggested a measure of caregiving-­related skills could also be included. Importantly, it appeared that completing the selected measures gave participants an opportunity to explicitly reflect on their lives and well-being. Paula reflected:

It was very good, especially the job satisfaction. It helped me reflect on how my life was at that moment. Which was very nice. And if there was something that was a little bit difficult, then you were like ‘Oh yeah, I can still do this’. (Paula, mentor)

All outcome measures showed high internal consistencies (GSES: 0.85–0.92; JSS 0.91–0.93; SWLS 0.83–0.89; PSS: 0.80–0.94) at all timepoints, suggesting high scale reliability of these measures. With respect to the outcome measures, convergent and discriminant validity, using Mukaka’s guidelines (Mukaka, 2012), one estimated correlation was high (JSS and SWLS at 6 months; 0.74), with another two considered moderate (JSS and GSES at 6 months, 0.55; and SWLS with GSES at 6 months, 0.587); all other correlations were either low or negligible (Supplementary Table 1). No measure was significantly correlated with PSS, and all estimated correlations with PSS were low. Aside from PSS, the strongest correlations were evidenced at baseline and 6-month assessments, where estimated correlations between the other three measurements pairwise were both positive and statistically significant. Aside from PSS, no correlation was significantly different from zero at the 3-month assessment, and all 3-month assessment correlations were smaller in magnitude than at baseline and 6-month assessments.

### Participation impact and exploratory analysis of outcomes

The majority of participants (mentees and mentors) reported experiencing a range of benefits and indicated they would strongly recommend this program to their colleagues. Many mentees, but also mentors, reported becoming more confident and less stressed at work and as a person in general, even those with many years of experience. They also argued the program filled a gap in support offered to caregivers, and especially those who were new to the job, or new immigrants. Stacey, who became very emotional during the interview, talked about her colleagues noticing her improvements:

It really helped me with my job. Like I said, I went to become a caregiver with no experience, and I was struggling. But after this, I’m so much more confident at my job. I am able to have a break and have my lunch. After this, I thought I can do this, I can do anything! (Stacey, mentee)

Not everyone was able to reach their program goals. However, for most mentees, taking part in the program appeared to drive their motivation and inspiration. Some were able to complete tasks they had delayed for months or years, for example, a caregiving-related qualification, a language exam, or starting a new project. Others were nominated for the caregiver of the year award or negotiated a pay rise. Mike used the program to get his overseas nursing qualification recognized in NZ:

The programme was absolutely life changing when it comes to creating your goals and someone helping you. And assisting you with your life. Especially foreigners, migrants coming from other countries. It’s a really great help for them as well. Because there is a mentor who will be able to guide them with their lives in New Zealand, and setting goals and a career. (Mike, mentee)

Exploratory outcomes analysis showed participants’ scores on all outcome measures trended in the expected direction (increases in GSES, JSS, SWLS; decrease in PSS; Table 4). Effect sizes between baseline and 6 months were very small to medium (PSS: 0.03; GSES: 0.11; JSS: 0.15; SWLS: 0.37). The change in SWLS at 6 months was statistically significant at the 5% level (*p* = .037) and was the only outcome to reach that threshold. Notably, the three remaining measures remained stable with some strong correlations with SWLS, indicating that a change in SWLS is unlikely to be attributable to regression to the mean.

**Table 4.** Changes in outcome measures.

Measures	Time 1 (*n *= 25)		Time 2 (*n *= 22)		Time 3 (*n *= 21)	
	Mean	*SD*	Mean	*SD*	Mean	*SD*
**GSES**	32.3	4.0	32.2	4.3	32.9	4.4
**JSS**	39.2	8.4	37.8	8.5	39.5	7.4
**PSS**	21.2	3.8	21.0	3.7	21.4	2.5
**SWLS**	24.5	7.4	25.6	6.0	27.4	6.1

*Note*. GSES = General Self-Efficacy Scale; JSS = Generic Job Satisfaction Scale; *n* = number of completed assessments; PSS = Perceived Stress Scale.

### Future definitive study

In general, participants expressed interest in taking part in a future definitive study of the program’s effectiveness. However, to some, being in the control group appeared “challenging,” and they wanted to know “what’s in it for me?.”

As noted, the change in SWLS was the only one to reach statistical significance, and it corresponded to approximately a 10% increase in the total score. Given the positive reports about the impact of the program on participants’ lives, the increase in SWLS scores arguably reflects a materially meaningful change.

We set the type 1 error probability at α=0.05 against a two-sided alternative and target 1-β=0.8 power to detect an improvement of 3 points on the score. We also assume unequal variances, corresponding to the standard deviations at Times 1 and 3 (Table 4) for the control and the intervention arms, respectively. Planning to adjust for baseline SWLS, which holds a 0.457 Pearson correlation with SWLS at 6 months, a parallel group study would need to recruit at least 77 and 53 mentees in the control and the intervention arms, respectively, bringing the overall target recruitment to 130 mentees. Given that only 13 of the 20 consented mentees completed the program, we recommend setting the recruitment target above 130 participants. A cluster effect from aged care providers would increase this sample size.

A less evidentially compelling design that might however encourage retention would be a pre–post study. In this instance, estimating the standard deviation of the difference in SWLS between Times 3 and 1 at 7.12, the study would require at least 47 mentees taking part.

Another approach would be to use a stepped-wedge cluster randomized trial design. This design is applicable to studies with heterogeneous populations, and would address the barrier that the traditional controlled trials pose, where only half of the participants receive the intervention. However, this approach requires recruitment of whole facilities and this has proven unfeasible in our experience of researching the NZ aged care context to date.

## Discussion and implications

The aim of this study was to investigate the feasibility and acceptability of the proposed intervention and to provide data required to plan a future definitive study. The current study found that the WeCare Mentoring Program was a feasible and acceptable intervention. The recruitment target was reached within the originally proposed 3-month timeframe. Data collection procedures were practical and convenient, with no missing data for any of the outcome measures. Participants (both mentees and mentors) reported positively on their experience of taking part. Importantly, mentees in the study included both less experienced and longer-serving aged care workers (0–11 years of experience), reflecting the program’s inclusive design for anyone motivated to engage in personal and/or professional development. All participants (mentees and mentors) found the program appealing and relevant and reported that the duration and frequency of mentoring sessions were appropriate, and the online format acceptable. Participants claimed it can address a range of issues affecting them in their daily lives as support workers but also as partners, parents, and/or immigrants. They appreciated the privacy and confidentiality the program format enabled. All participants reported noticing a range of improvements in their well-being and their caregiving-related skills. Participants proposed changes which could improve the experience further (Table 5) but were overwhelmingly in support of the program. They would recommend, or already have recommended, this program to other support workers.

**Table 5.** Final refinements to the proposed intervention protocol.

Aspect of the protocol	Description
**Recruitment**	Recruit primarily via bulk messaging, e.g., via e-mail Partner with organizations that are trusted by support workers, e.g., staff unions Facilitate snowball sampling Incorporate the reasons for taking part as the key drawcards in recruitment advertisements In all recruitment documents, clarify that part-time and casual workers are also invited
**Outcome measures**	Replace the GSES with a self-efficacy measure specific to support workers Remove one of the satisfaction measures; given a job context-specific measure of self-efficacy is recommended, retaining the SWLS appears reasonable Add a measure of participant satisfaction with mentee/mentor match Measure the effect of taking part in the program on the quality of care
**Training**	Develop video materials to accompany the program manual
**Mentee/mentor matching**	Provide more background about mentors, focusing specifically on their key areas of expertise Provide information on participants’ preferences regarding meeting frequency
**Mentoring relationship**	Work with aged care providers to allow participation during staff’s paid study time Consider developing a one-stop online platform for the program Develop means to further enhance adaptability of the program to support workers’ schedules, e.g., online meeting scheduling system, automated reminders, direct messaging Remove the ‘session duration of 30 to 60 minutes’ recommendation, but continue monitoring the session duration Clarify the purpose of goal setting to reflect its use as a means of providing focus and structure rather than as an outcome measure Encourage mentors to message their mentees between mentoring sessions to facilitate engagement

*Note*. SWLS = Satisfaction with Life Scale.

In the current study, the recruitment target was reached within the originally proposed recruitment period. Even though the “presentations” response rate was seven percent, the response rate from other recruitment approaches (poster and staff union email) was much lower, with the overall response rate being only 4%. This is perhaps unsurprising given that participant recruitment has been identified as the most difficult aspect of intervention studies (Hughes-Morley, 2017) and particularly so in underrepresented populations (George et al., 2014). Furthermore, unlike most intervention studies for support workers, current participants were asked to take part in their own time. Participants in the current study expressed a preference for being able to conduct their mentoring sessions during work hours and reported concerns around the confidentiality of their participation. Trust and convenience of participation were identified as important factors influencing recruitment (George et al., 2014). To increase the convenience of participation, it will be worth negotiating with the aged care organizations to allow their workers to use their study time to take part in the program. As reported by participants, it is relatively common for employers to offer 1–2 hr of paid time per week for workers to study for their qualifications. To increase trust, it will be important to advertise the study through trusted channels (e.g., staff unions, other support workers, enrolled participants). Several current participants reported telling their colleagues about the program and reported that they wanted to take part. Careful consideration of these and other methods to facilitate future study recruitment is recommended.

Another area worth specific consideration relates to measuring this program’s outcomes. Nearly all mentees and some mentors talked about how taking part in this program helped them become better at caregiving. This improvement affected technical aspects of the job as well as personal skills and attitudes. This is an important finding because it provides initial evidence that the holistic focus of the program (i.e., being able to set goals relating to any aspect of one’s life) can help participants become better workers. This can lead to improvements in quality of care for aged care recipients, and a range of other organization-level gains (e.g., lower staff turnover, lower absenteeism), thus making this program attractive not only to support workers but also to their employers. Previous research on measuring training needs and competencies in the closely related field of nursing proposed that measurement should focus on factors such as clinical skills and knowledge but also interpersonal and problem-solving skills, moral sensibility, commitment, and compassion (Han et al., 2014; Needleman et al., 2007). Closely related to these factors is self-efficacy, which is underpinned by a worker’s professional identity and competence (Caruso et al., 2016) and is an important predictor of performance, job satisfaction, and well-being (Caruso et al., 2016; Cheraghi et al., 2009). Importantly, task-specific self-­efficacy has been argued as likely more sensitive to change than generalized self-efficacy (Smith et al., 2006; Stajkovic & Luthans, 1998). The future study should include an instrument with the ability to measure self-efficacy as it relates to the specific reality of being a support worker. As no such tool has been proposed to date, an adaptation of one of the existing instruments used in the nursing research may be the best possible option; for example, the Nursing Profession Self-Efficacy Scale (Caruso et al., 2016) or the Self-Efficacy Clinical Performance Scale (Cheraghi et al., 2009), or the National Dementia Care Workforce Surveys (https://www.ndws.org/surveys-and-data/survey-questionnaires). Adapting these instruments would require a review of their content and acceptability and an evaluation of validity and reliability for use with support workers in NZ.

Since the time of this study in 2019, the field and accessibility of online mentoring have made further developments (Weinberg & Scandura, 2024). Virtual engagement has been significantly normalized, enhancing the feasibility and acceptability of online programming; video conferencing software has been improved, and access to a stable internet connection has been broadened (Weinberg & Scandura, 2024). Moreover, there have been early trials of AI-assisted mentoring, showing this approach holds a lot of potential but also carries significant risks and limitations (Graßmann & Schermuly, 2021). These developments point to several future research opportunities, such as investigating the role of emerging technologies (e.g., the use of AI), exploring how this and similar programs could work across other workforce groups and other settings (e.g., aged care nurses, disability support workers, home support workers). The content and format of the proposed intervention can be tailored to the specific needs and preferences of each user, and as such, it could be relatively easily adapted to other settings and populations.

### Limitations

This study was not designed to determine the effectiveness of the proposed intervention. Even though one of the outcome scores (SWLS) changed in the expected direction and the participant reports suggest the program was effective, further testing using an appropriate design is required to establish the effectiveness of this intervention.

We were guided by the Medical Research Council Framework to develop and evaluate the intervention. We found that the framework was helpful in structuring key phases, but it could be further strengthened by explicit integration of implementation frameworks, strategies, and outcomes. Our approach focused primarily on feasibility and acceptability, and future work could extend this by more explicitly embedding implementation science concepts to enhance the rigor and generalizability of future research.

The proposed intervention requires its users to have access to the internet and a basic level of digital literacy. As such, not everyone might be able, or willing, to use the intervention. Nonetheless, no one was excluded from taking part because of having no internet access, and/or insufficient digital literacy level. On the contrary, participants reported that taking part in the program was easy even for those who considered themselves to have very limited computer skills.

Finally, the in-person recruitment was conducted only at facilities that agreed to have their staff be approached about this study. Two group providers (both managing over 20 residential aged care facilities) declined to take part, stating potential cost as the reason. Support workers employed by those very organizations may also be those who urgently need access to interventions like this study. Including all support workers, regardless of their employers’ funding ability, should be the key focus of the future definitive study.

## Conclusion

The WeCare Mentoring Program is a feasible and acceptable intervention to improve health and well-being outcomes for support workers. It incorporates international evidence on strategies to support this workforce with perspectives from a range of local stakeholders in NZ. Its users reported improvements in a range of areas of their professional and personal lives. Its key strengths include:

- Having a flexible and boundary-free format, allowing support workers to take part at a time and location that suits them
- Having a holistic focus, allowing its users to concentrate on topics that are important and meaningful to them
- Being free of cost to aged care organizations, addressing the economic barrier
- Being built on an evidence-based concept of peer mentoring, which has been shown to improve a range of outcomes across many workforce groups

A range of refinements were proposed to enhance the program’s feasibility. The next step is to test the intervention’s effectiveness in a definitive randomized controlled trial or quasi-experimental study. If effective, this program will offer a much-needed support to people who have been historically undervalued and are experiencing increasingly difficult working conditions. Better support for these workers is likely to lead to better health outcomes for them and the people they care for.

## Supplementary material

Supplementary data are available at *Innovation in Aging* online.

## Supplementary Material

igaf094_Supplementary_Data

## Data Availability

Data are available from the first author upon reasonable request. This study was not preregistered.
