# Supplementary material for: Feasibility and acceptability of WeCare Mentoring, an online peer mentoring program for aged care support workers
Source: Innov Aging. 2025 Sep 10;9(10):igaf094. doi: 10.1093/geroni/igaf094 (PMC12588540; doi:10.1093/geroni/igaf094)
Supplement: igaf094_Supplementary_Data [file igaf094_supplementary_data.zip › innage suppl Czuba, Vandal, & Kayes.docx]

***Innovation in Aging* Supplementary Material: Czuba, Vandal, & Kayes. Feasibility and acceptability of WeCare Mentoring, an online peer mentoring programme for aged care support workers.**

**Supplementary Methods**

**Section 1. Recruitment methods description**

1. Poster – posters were presented in staff areas (e.g., lunch rooms) and included a brief overview of the research and researcher contact details.
2. Presentation and pamphlet– presentations were delivered in person by KC at staff meetings at workplaces, and provided a brief overview of the study; each attendee received a pamphlet with the study overview and researcher contact details.
3. Staff union email invitation – a large staff union emailed its members inviting them to contact the researcher if they were interested in taking part; the participant information sheet was attached to the email.

**Section 2. Outcome measures description**

- Satisfaction with Life Scale (SWLS) (Diener, Emmons, Larsen, & Griffin, 1985): five-item scale measuring respondents’ judgments (e.g., ‘In most ways my life is close to my ideal’), using a seven-point rating scale ranging from 1 (strongly disagree) to 7 (strongly agree).
- Generic Job Satisfaction Scale (JSS) (Macdonald & Maclntyre, 1997): a ten-item scale of universal job satisfaction (e.g., ‘I feel good about my job’), using a five-point rating scale ranging from 1 (strongly disagree) to 5 (strongly agree).
- Perceived Stress Scale (PSS) (Cohen, Kamarck, & Mermelstein, 1994): a ten-item scale measuring the degree to which situations in one’s life are appraised as stressful (e.g., ‘In the last month, how often have you been upset because of something that happened unexpectedly?’), using a five-point rating scale ranging from 0 (never) to 4 (very often).
- General Self-Efficacy Scale (GSES) (Schwarzer & Jerusalem, 1995): a ten-item scale measuring respondents’ judgements (e.g., ‘I can always manage to solve difficult problems if I try hard enough’), using a four-point rating scale ranging from 1 (not at all true) to 4 (exactly true).

**Section 3. Interview guide - Mentee**

*Participants will be reflecting on their experience of participating in the WeCare Mentoring intervention.*

**Start audio-recording**

**Training**

Can you tell me a bit about your thoughts on the training manual?

Can you tell me a bit about your thoughts on the Skype briefing?

Did you feel the training prepared you for your first meeting? Using Skype? Scheduling meetings? Setting goals for yourself?

Was there anything missing in the training that might have been helpful?

**Mentor matching**

What did you think about the mentor matching process? What did you think when you met your mentor?

**Mentoring intervention**

How did you find the mentoring session?

How did you manage engaging in a mentoring session with your mentor?

How did you find completing the meeting reports?

**Overall experience**

Can you tell me a bit more about your impressions regarding the intervention?

In general, how relevant is this intervention to you?

What did you like about the overall process? What did you not like?

Did you complete or enrolled into any training as a result of this intervention?

In what ways has taking part in this intervention affected you?

What affected your engagement in this intervention?

**Stop audio-recording**

**Section 4: Interview guide - Mentor**

*In this semi-structured Interview, you will be reflecting on your experience of participating in the e-mentoring programme.*

**Start audio-recording**

*The main aim of this interview is on perceived acceptability and other feasibility aspects of the e-mentoring programme.*

**Acceptability and suitability of recruitment protocols**

How did you find out about the study?

How was the recruitment process for you?

What did you like about the process? What did you not like?

**Acceptability and suitability of the programme**

Can you tell me a bit more about your impressions regarding the programme? What did you like? Not like?

What did you find most useful? Least useful?

How could the programme be improved?

In general, how relevant is this programme for you?

What could affect people’s engagement in this programme?

What did you think about the programme manual?

What characteristics do you think make a good mentor?

Tell me about your mentee. What did you like/not like about their approach?

**Preliminary responses to the programme**

In what way did this programme affect you? How did it affect you as a support worker?

What did you think about the frequency of your sessions? What about duration?

Would you recommend this programme to others? Why? Why not?

**Acceptability and suitability of data collection procedures and outcome measures**

How did you go with completing the online surveys?

What did you think about the format and wording of the items?

Can you tell me about any difficulties you experienced completing the surveys?

What did you think about the constructs we have selected to measures? Where these surveys able to capture the impact of the programme on you? What other constructs would you recommend focusing on?

**Stop audio-recording**

**Section 5: Quantitative indicators related to the study processes**

Response rate was calculated as the number of people who enquired about the study divided by the total number of people who were likely to have received the study invitation. Consenting rate was calculated as the total number of eligible people who consented to participation in the study divided by the total number of eligible people who enquired. The number of days from the invitation to consenting reflects the time required to recruit a mentor and a mentee. Follow-up rate was expressed as the number of completed outcome surveys at each time point (baseline, three, and six months) divided by the total number of active study participants at that time. Session completion rate was expressed as the number of completed mentoring sessions divided by the total number of planned sessions. Fidelity rate was expressed as the number of sessions that met the intervention fidelity recommendations (30-60-minutes mentoring sessions; goal setting and reviewing; and post-session reporting/reflection) by the total number of completed sessions. Frequency of mentoring sessions was expressed as the average number of sessions conducted by a mentee, divided by the time between the first and last session (in days).

**Supplementary Table 1: Outcome measure scores correlations.**

Pearson’s correlations between outcome measures at baseline, three, and six-month assessments.

| **Outcome 1** | **Outcome 2** | **Assessment** | **Correlation Coefficient** | **Lower 95%CI** | **Upper 95%CI** | **St Error** |
| --- | --- | --- | --- | --- | --- | --- |
| JSS | SWLS | 1 | 0.30 | -0.11 | 0.62 | 0.18 |
| JSS | SWLS | 2 | 0.38 | -0.05 | 0.69 | 0.18 |
| JSS | SWLS | 3 | 0.74 | 0.45 | 0.89 | 0.10 |
| JSS | GSES | 1 | 0.46 | 0.08 | 0.74 | 0.16 |
| JSS | GSES | 2 | 0.24 | -0.30 | 0.60 | 0.20 |
| JSS | GSES | 3 | 0.55 | 0.16 | 0.79 | 0.15 |
| JSS | PSS | 1 | -0.07 | -0.45 | 0.34 | 0.20 |
| JSS | PSS | 2 | 0.16 | -0.28 | 0.54 | 0.21 |
| JSS | PSS | 3 | -0.08 | -0.50 | 0.36 | 0.22 |
| SWLS | GSES | 1 | 0.44 | 0.05 | 0.71 | 0.16 |
| SWLS | GSES | 2 | 0.14 | -0.30 | 0.53 | 0.21 |
| SWLS | GSES | 3 | 0.59 | 0.21 | 0.81 | 0.14 |
| SWLS | PSS | 1 | -0.15 | -0.51 | 0.26 | 0.20 |
| SWLS | PSS | 2 | 0.30 | -0.14 | 0.64 | 0.19 |
| SWLS | PSS | 3 | 0.06 | -0.38 | 0.48 | 0.22 |
| GSES | PSS | 1 | -0.05 | -0.44 | 0.35 | 0.20 |
| GSES | PSS | 2 | -0.16 | -0.54 | 0.28 | 0.21 |
| GSES | PSS | 3 | -0.06 | -0.48 | 0.38 | 0.22 |
